# Endothelin A Receptor Antagonism Enhances Inhibitory Effects of Anti-Ganglioside GD2 Monoclonal Antibody on Invasiveness and Viability of Human Osteosarcoma Cells

**DOI:** 10.1371/journal.pone.0093576

**Published:** 2014-04-11

**Authors:** Bo Liu, Yi Wu, Yu Zhou, Dan Peng

**Affiliations:** 1 Department of Orthopaedics, The Second Xiangya Hospital, Central South University, Changsha, Hunan, P. R. China; 2 Hunan Provincial Health Bureau, Changsha, Hunan, P. R. China; Inserm U606 and University Paris Diderot, France

## Abstract

Endothelin-1 (ET-1)/endothelin A receptor (ETAR) signaling is important for osteosarcoma (OS) progression. Monoclonal antibodies (mAbs) targeting ganglioside GD2 reportedly inhibit tumor cell viability independent of the immune system. A recent study suggests that ganglioside GD2 may play an important role in OS progression. In the present study, we for the first time explored the effects of anti-GD2 mAb alone or in combination with ETAR antagonist on OS cell invasiveness and viability. Human OS cell lines Saos-2, MG-63 and SJSA-1 were treated with control IgG (PK136 mAb, 50 µg/mL), anti-GD2 14G2a mAb (50 µg/mL), selective ETAR antagonist BQ123 (5 µM), or 14G2a (50 µg/mL)+BQ123 (5 µM). Cells with knockdown of ETAR (ETAR-shRNA) with or without 14G2a mAb treatment were also tested. Cells treated with selective phosphatidylinositide 3-kinase (PI3K) inhibitor BKM120 (50 µM) were used as a positive control. Our results showed that BQ123, ETAR-shRNA and 14G2a mAb individually decreased cell invasion and viability, matrix metalloproteinase-2 (MMP-2) expression and activity, PI3k activity, and phosphorylation at serine 473 (ser473) of Akt in OS cells. 14G2a mAb in combination with BQ123 or ETAR-shRNA showed significantly stronger inhibitory effects compared with each individual treatment. In all three cell lines tested, 14G2a mAb in combination with BQ123 showed the strongest inhibitory effects. In conclusion, we provide the first in vitro evidence that anti-ganglioside GD2 14G2a mAb effectively inhibits cell invasiveness, MMP-2 expression and activity, and cell viability in human OS cells. ETAR antagonist BQ123 significantly enhances the inhibitory effects of 14G2a mAb, likely mainly through inhibiting the PI3K/Akt pathway. This study adds novel insights into OS treatment, which will serve as a solid basis for future in vivo studies on the effects of combined treatment of OS with anti-ganglioside GD2 mAbs and ETAR antagonists.

## Introduction

Osteosarcoma (OS) is the most frequent primary bone malignancy and the eighth most common type of cancer among children, comprising 2.4% of all malignancies in pediatric patients and approximately 35% of all bone cancers [1]. OS is a devastating disease, characterized by high local aggressiveness and a tendency to metastasize to the lungs and distant bones. The cure rate of OS is approximately 65% for patients with localized diseases. When presenting with metastases at the time of diagnosis, the survival rate is 25% [2], [3]. Despite modern multidisciplinary treatments including chemotherapy and surgery, the 5-year survival rate of osteosarcoma patients remains 60%–70% [1]. Hence, there is an urgent need to develop novel approaches to treat OS patients, particularly, to identify and confirm potential therapeutic targets involved in OS progression.

Gangliosides are glycosphingolipids carrying one or several sialic acid residues. They are essentially located on the outer leaflet of the plasma membrane in microdomains named “glycosynapses”, where they can interact with transmembrane receptors or signal transducers involved in cell proliferation and signaling [4]. The tumor-associated ganglioside GD2 is an attractive target for immunotherapy. While its expression in normal tissue is restricted to the central nervous system and peripheral nerves, it is strongly detectable on neuroblastoma and on most melanoma lesions [5]. Additionally, it is found on sarcoma, glioma and in approximately 50%–100% of small cell lung cancers where it is associated with enhanced cell viability and invasive activity [5]. Due to its distribution pattern, GD2 has been chosen as a target for monoclonal antibody (mAb) therapy. Early clinical trials indicated certain efficacy especially in the treatment of neuroblastoma [6]. mAbs targeting tumor-associated gangliosides reportedly may inhibit tumor cell viability by means of immunological mechanisms such as antibody-dependent cell-mediated cytotoxicity, complement-dependent cytotoxicity, and the anti-idiotypic network [7]. However, there has been a growing number of evidence that GD2-specific antibodies may exhibit anti-viability effects without involvement of the immune system [7]. It has been shown that anti-GD2 mAb is capable of decreasing viability of human neuroblastoma cells in a dose-dependent manner [8]. A recent study has shown that GD2 is highly expressed in OS tissues and cell lines. In addition, OS tissue obtained at the time of disease recurrence shows higher intensity of GD2 staining compared with samples obtained at initial biopsy and definitive surgery [9]. The findings suggest that ganglioside GD2 may play an important role in OS progression.

Endothelin-1 (ET-1), a potent vasoconstrictor initially isolated from endothelial cells, is involved in a wide range of cancer-relevant processes, such as inhibition of apoptosis, matrix remodeling, and metastases [10]. ET-1 and ET A receptor (ETAR) are expressed in OS tissue and cells [10], [11]. Previous studies suggest that ET-1/ETAR signaling is important for OS progression and metastasis [10]–[12]. Zhao et al. reported that ET-1/ETAR signaling could promote OS cell invasion and survival [10]. Felx et al. reported that ET-1 could promote OS cell invasion by inducing the synthesis of matrix metalloproteinase-2 (MMP-2) through ETAR [11]. Li et al. showed that ETAR was critical for OS pulmonary metastasis in an orthotopic xenograft OS model [12].

The phosphatidylinositol 3-kinase (PI3K)/Akt pathway reportedly plays an important role in OS cell invasiveness and viability [13]–[16]. ET-1 has been reported to activate the PI3K/Akt pathway via the ETAR [17]. The PI3K/Akt pathway is also involved in ganglioside GD2-induced tumorigenicity and aggressiveness of breast cancer cells [18]. Thus, we hypothesized that blocking ET-1/ETAR signaling and GD2 may have combinatorial effects on OS cell invasiveness and viability. In the present study, we explored the effects of anti-GD2 mAb alone or in combination with ETAR antagonist on OS cell invasiveness and viability in vitro.

## Materials and Methods

### Cells lines and reagents

Saos-2, MG-63 and SJSA-1 human OS cell lines and PK136 hybridoma cell line were purchased from the American Type Culture Collection (Manassas, VA, USA). ETAR shRNA lentiviral particles (sc-39960-V), control shRNA lentiviral particles-A (sc-108080), selective phosphatidylinositide 3-kinase (PI3K) inhibitor BKM120 (sc-364437A), anti-ETAR (sc-21193) antibody, anti-matrix metalloproteinase-2 (MMP-2) antibody (sc-53630), anti-Akt (5C10) (sc-81434) and anti-P-Akt (ser473) (sc-101629) antibodies, and a mouse anti-ganglioside GD2 mAb (14G2a) (sc-53831) were purchased from Santa Cruz Biotechnology (Santa Cruz, CA, USA). A mouse isotype-matched control PK136 mAb for 14G2a were purified from hybridoma culture supernatants using the Hi-Trap Protein G HP column (GE Healthcare Life Sciences, Shanghai, China) according to the manufacturer's protocol. The SensoLyte 520 MMP-2 Assay Kit (71151) was purchased from AnaSpec (Fremont, CA, USA). The QCM ECMatrix 24-well (8 µM) Fluorimetric Cell Invasion Assay kit (ECM554) was purchased from Chemicon (Millipore, Billerica, CA, USA). The ET-1 ELISA kit and the Methlythiazoletetrazolium (MTT) cell viability assay kit was purchased from R&D Systems (Minneapolis, MN, USA). The PI3K Activity ELISA kit (K-1000s) was purchased from Echelon Biosciences (Salt Lake City, UT, USA). Selective ETAR antagonist BQ123 was purchased from Sigma (St. Louis, MO, USA).

### Scatchard analysis

Anti-GD2 14G2a mAb was labeled with I-125 (PerkinElmer, Billerica, MA) using the iodogen method, and were purified on a Sephadex PD10 column (Pharmacia Biotech, Piscataway, NJ, USA). The binding assays for Scatchard analysis were performed as previously described [8]. Briefly, serial dilutions of labeled antibody were incubated for 1 h at 4°C with 10^6^ cells. Cell-bound radioactivity was separated from free antibody by centrifugation through a dibutyl phthalate oil cushion in microfuge tubes. Pellet cells and supernatant activity were then separately measured using a gamma counter (PerkinElmer, Beijing, China). Non-specific binding, defined as the bound ^125^I-labeled antibody in the presence of a 100-fold excess of unlabeled antibody, was subtracted at each concentration of labeled antibody. The binding data were analyzed on Prism software (GraphPad Prism Software, La Jolla, CA, USA) according to a one-site equilibrium binding equation.

### Lentiviral Transduction

The ETAR shRNA lentiviral particles contain expression constructs encoding target-specific 19–25 nt (plus hairpin) shRNA designed to specifically knockdown *ETAR* gene expression. The control shRNA lentiviral particles contain a scrambled shRNA sequence that will not lead to specific degradation of any cellular mRNA, and was used as a negative control for ETAR shRNA lentiviral particles. Lentiviral transduction was performed in Saos-2, MG-63 and SJSA-1 cells. Pools of stable transductants were generated via selection with puromycin (5 µg/ml) by the manufacturer's protocol (Santa Cruz Biotechnology).

### Cell invasion assay

In vitro cell invasion assays were performed with the QCM ECMatrix 24-well (8 µM) Fluorimetric Cell Invasion Assay kit (Chemicon; Millipore) according to the manufacturer's instructions [19], [20]. The kit used an insert polycarbonate membrane with an 8 µM pore size. The insert in the invasion kit was coated with a thin layer of ECMatrix. Cell invasion was determined by fluorescence. Each experiment was repeated for three times in duplicates.

### Real-time quantitative reverse transcription PCR

RNA was prepared from cells using TRIzol reagent followed by purification with TURBO DNA-free system (Ambion, Austin, TX, USA). The cDNAs were synthesized using SuperScript II reverse transcriptase (Invitrogen, Carlsbad, CA, USA). Real-time quantitative PCR was performed on the LightCycler thermal cycler system (Roche Diagnostics, Indianapolis, IN, USA) using SYBR Green I kit (Roche) as described by the manufacturer. The results were normalized against that of housekeeping gene glyceraldehyde-3-phosphate dehydrogenase (GAPDH) in the same sample. The primers used are as follows: for MMP-2, 5′-CAAGTTTCCATTCCGCTTC-3′ (forward) and 5′-GTTCCCACCAACAGTGGACA-3′ (reverse); for GAPDH, 5′-GACTCATGACCACAGTCCATGC-3′ (forward) and 5′-AGAGGCAGGGATGATGTTCTG-3′ (reverse). Each experiment was repeated for three times in duplicates.

### Immunoassays

The secreted ET-1 levels in cell culture supernatants were determined using an ET-1 ELISA kit. In brief, cells were grown to confluence in 10-cm dishes in RPMI 1650 medium supplemented with 10% FBS, followed by replacing the medium with serum-free medium and further incubation for 16 h. The cell culture supernatants were collected for ELISA according to the manufacturer's instructions (R&D Systems). ELISA-detected ET-1 concentrations were normalized against cell number (per 10^6^ cells). Each ELISA experiment was repeated for three times in duplicates. In Western blot analyses, cells were treated with control IgG (PK136 mAb, 50 µg/mL), 14G2a (50 µg/mL), BQ123 (5 µM), 14G2a (50 µg/mL)+BQ123 (5 µM), or BKM120 (50 µM) for 48 hr and then dissolved in 250 µl of 2× SDS loading buffer (62.5 mM TrisHCl, pH 6.8, 2% SDS, 25% glycerol, 0.01% bromphenol blue, 5% 2-mercaptoethanol), and incubated at 95 °C for 10 min. Equal amount of proteins for each sample were separated by 10% SDS-polyacrylamide gel and blotted onto a polyvinylidene difluoride microporous membrane (Millipore, Billerica, MA, USA). Membranes were incubated for 1 h with a 1/500 dilution of anti-MMP-2, anti-P-Akt (ser473) or anti-Akt antibody, and then washed and revealed using secondary antibodies with horseradish peroxidase conjugate (1/5000, 1 h). Peroxidase was revealed with a GE Healthcare ECL kit (Shanghai, China). Three independent experiments were performed for each Western blot analysis.

### MMP-2 activity assay

MMP-2 activity was measured with the SensoLyte 520 MMP-2 Assay Kit (AnaSpec) according to the manufacturer's instructions [21], [22]. The supernatants were collected and then incubated with 4-aminophenylmercuric acetate (AMPA) and MMP-2 substrate. The fluorescence intensity at Ex/Em Wave lengths of 490 nm/520 nm were used as a measure of MMP-2 activity. Each experiment was repeated for three times in duplicates.

### MTT cell viability assay

In vitro cell viability was determined with the MTT cell viability assay kit as described by the manufacturer (R&D systems). Briefly, cells were cultured at 15×10^3^ cells per well in 96-well tissue culture plates and incubated at 37°C for 24 or 48 hr with or control IgG (PK136 mAb, 50 µg/mL), 14G2a (50 µg/mL), BQ123 (5 µM), 14G2a (50 µg/mL)+BQ123 (5 µM), or BKM120 (50 µM) for 24 or 48 hr. At the end of the culture period, cells were washed with PBS, the MTT reagents were added according to the manufacturer's recommendations, and the absorbance was measured at 570 nm using an ELISA plate reader. Viability of the control cells was defined as 100%. The inhibition rate of cell viability was calculated and shown as a percentage of the control cell viability. Each experiment was repeated for three times in triplicates.

### PI3K activity assay

PI3K activity was determined with the PI3K Activity ELISA kit (Echelon Biosciences) according to the manufacturer's instructions [23], [24]. For direct functional assessment of PI3K activity, PI3K was isolated by immunoprecipitation using an anti-PI3K antibody (Millipore, #06-195) to the p85 adapter subunit, and the ability of the co-precipitated catalytic p110 catalytic subunit to convert a standard PIP2 to PIP3 in a kinase reaction assessed by measuring the generated PIP3 by the ELISA kit. Each experiment was repeated for three times in duplicates.

### Statistical analysis

Statistical analyses were performed with SPSS for Windows 10.0. All data values were expressed as means ± SD. Comparisons of means among multiple groups were performed with one-way ANOVA followed by *post hoc* pairwise comparisons using Tukey's tests. A two-tailed *p*<0.05 was considered statistically significant in this study.

## Results

### Expression levels of ganglioside GD2, ETAR and ET-1 in human OS cell lines

**Figure 1:**
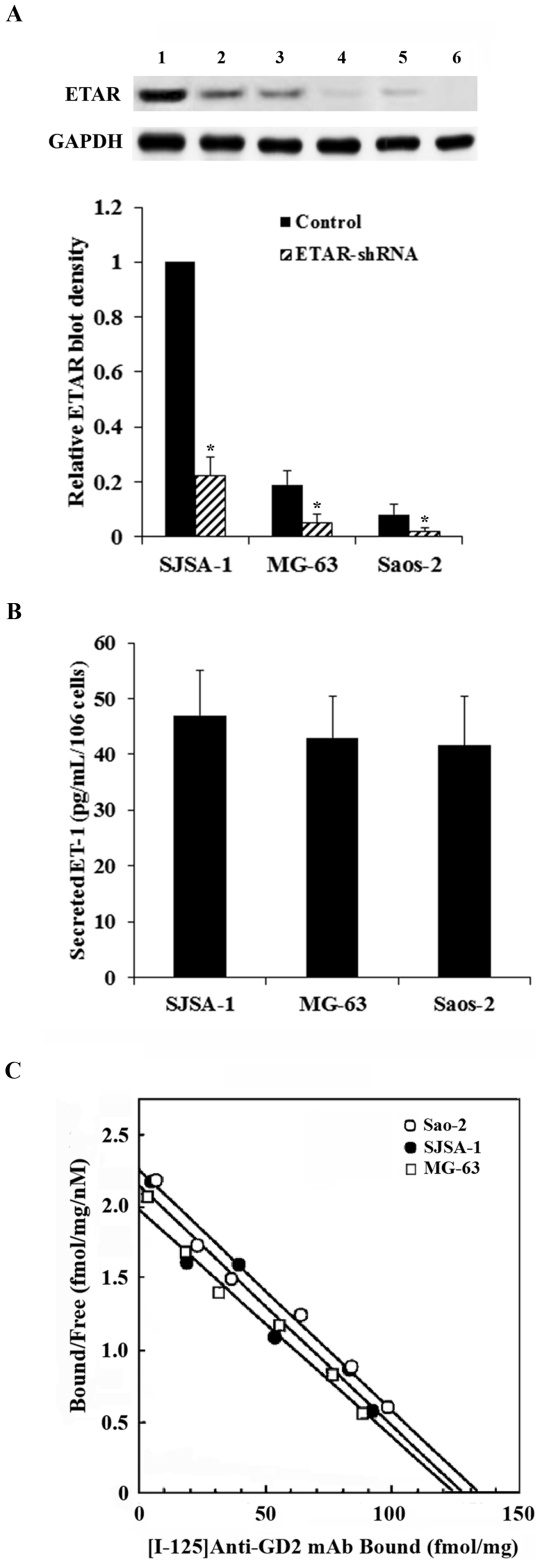
Endothelin-1 (ET-1) and ET A receptor (ETAR) expression levels and scatchard plot for anti-ganglioside GD2 14G2a monoclonal antibody (mAb) binding sites in human osteosarcoma (OS) cells. (*A*) ETAR levels were determined by Western blot analysis in Saos-2, MG-63 and SJSA-1 human OS cell lines. *Lane 1*, SJSA-1 cells; *lane 2*, SJSA-1 cells stably transduced with ETAR-shRNA; *lane 3*, MG-63 cells; *lane 4*, MG-63 cells stably transduced with ETAR-shRNA; *lane 5*, Saos-2 cells; *lane 6*, Saos-2 cells stably transduced with ETAR-shRNA. Glyceraldehyde-3-phosphate dehydrogenase (GAPDH) blotting was used as a loading control. Density of the ETAR blot was normalized against that of GAPDH to obtain a relative blot density, which was expressed as fold changes to the relative ETAR blot density of SJSA-1 control cells (designated as 1). Three independent experiments were performed for each Western blot analysis. Data values were expressed as Mean+SD. *p<0.05 vs. control. (*B*) Secreted ET-1 levels in cell culture supernatants were quantified using ELISA and normalized against cell number (per 10^6^ cells). Each ELISA experiment was repeated for three times in duplicates. Data values were expressed as Mean+SD. (*C*) Scatchard plot for binding of I-125-labeled anti-ganglioside GD2 14G2a mAb to OS cells.

We used Saos-2, MG-63 and SJSA-1 human OS cell lines as cell models in this study. Western blot analyses revealed significant differences in the ETAR expression level among the cell lines, with SJSA-1 cells expressing the highest level and Saos-2 expressing the lowest level of ETAR (Fig. 1*A*). We stably transduced the cells with ETAR-shRNA to knock down ETAR. As shown in Fig. 1*A*, the endogenous ETAR level was knocked down over 75% in all three cell lines. ELISA showed no significant differences in the secreted ET-1 level among the cell lines (Fig. 1*B*). Scatchard analysis using ^125^I-labeled mouse anti-ganglioside GD2 mAb (14G2a) revealed that the three cell lines had similar levels of 14G2a binding sites as follows: Saos-2, (52±8)×10^5^/cell; MG-63, (49±6)×10^5^/cell; SJSA-1, (51±9)×10^5^/cell. The results indicate that the three cell lines express similar levels of ganglioside GD2 on the cell surface (Fig. 1*C*).

### Effects of anti-ganglioside GD2 14G2a mAb alone or in combination with ETAR antagonist on OS cell invasiveness and MMP-2 expression

**Figure 2:**
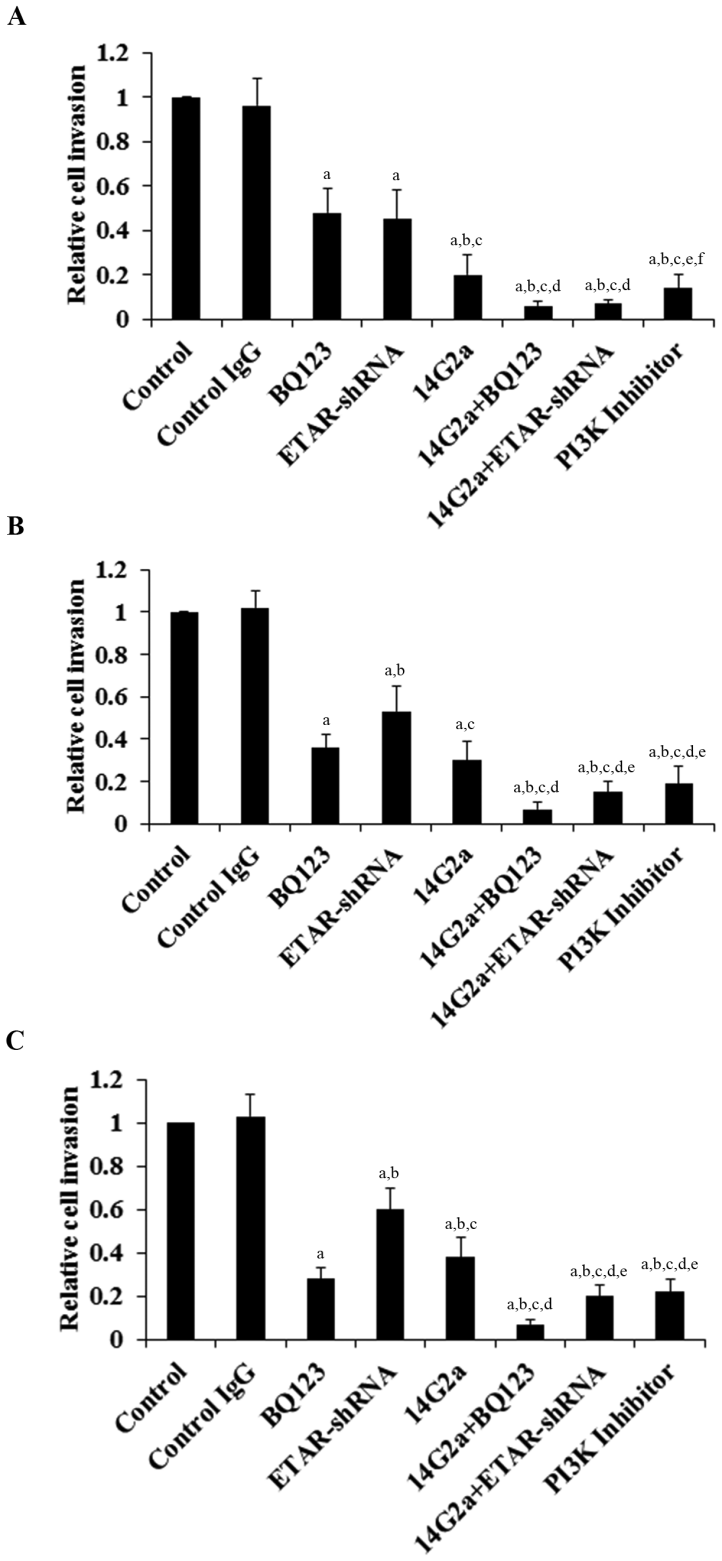
Effects of anti-ganglioside GD2 14G2a monoclonal antibody (mAb) alone or in combination with ET A receptor (ETAR) antagonist on osteosarcoma (OS) cell invasiveness. In vitro cell invasion assays were performed with a Fluorimetric Cell Invasion Assay kit (Chemicon; Millipore) in Saos-2 (*A*), MG-63 (*B*) and SJSA-1 (*C*) OS cells treated with control IgG (PK136 mAb, 50 µg/mL), 14G2a mAb (50 µg/mL), selective ETAR antagonist BQ123 (5 µM), and 14G2a (50 µg/mL)+BQ123 (5 µM) for 48 hours. Cells with knockdown of ETAR (ETAR-shRNA) with or without 14G2a mAb treatment were also tested. Cells treated with selective phosphatidylinositide 3-kinase (PI3K) inhibitor BKM120 (50 µM) was used as a positive control. Cell invasion was determined by fluorescence and shown as fold changes to that of the untreated control cells (designated as 1). Each experiment was repeated for three times in duplicates. Data values were expressed as Mean+SD. ^a^ *p*<0.05 vs. control or control IgG; ^b^ *p*<0.05 vs. BQ123; ^c^ *p*<0.05 vs. ETAR-shRNA; ^d^ *p*<0.05 vs. 14G2a; ^e^ *p*<0.05 vs. 14G2a+BQ123; ^f^ *p*<0.05 vs. 14G2a+ETAR-shRNA.

To explore the effects of anti-ganglioside GD2 mAb alone or in combination with ETAR antagonist on OS cell invasiveness, we conducted in vitro cell invasion assays. Among different anti-GD2 mAbs titrated, 14G2a at 50 µg/mL showed strongest inhibitory effects on OS cell invasion and viability in our pilot studies (data not shown) and thus was employed in this study. A mouse isotype-matched mAb purified from PK136 hybridoma culture supernatants was used as a control Ab/IgG for 14G2a. As shown in Fig. 2, Saos-2 (*A*), MG-63 (*B*) and SJSA-1 (*C*) cells were treated with control IgG (PK136 mAb, 50 µg/mL), 14G2a mAb (50 µg/mL), selective ETAR antagonist BQ123 (5 µM), and 14G2a (50 µg/mL)+BQ123 (5 µM) for 48 hours. Cells with knockdown of ETAR (ETAR-shRNA) with or without 14G2a mAb treatment were also tested. Cells treated with selective PI3K inhibitor BKM120 (50 µM) was used as a positive control [25]. Fig. 2 shows that BQ123, ETAR-shRNA and 14G2a mAb individually decreased OS cell invasion. 14G2a mAb in combination with BQ123 or ETAR-shRNA showed significantly stronger inhibitory effects compared with each individual treatment. In all three cell lines tested, 14G2a mAb in combination with BQ123 showed the strongest inhibitory effects on OS cell invasion.

**Figure 3:**
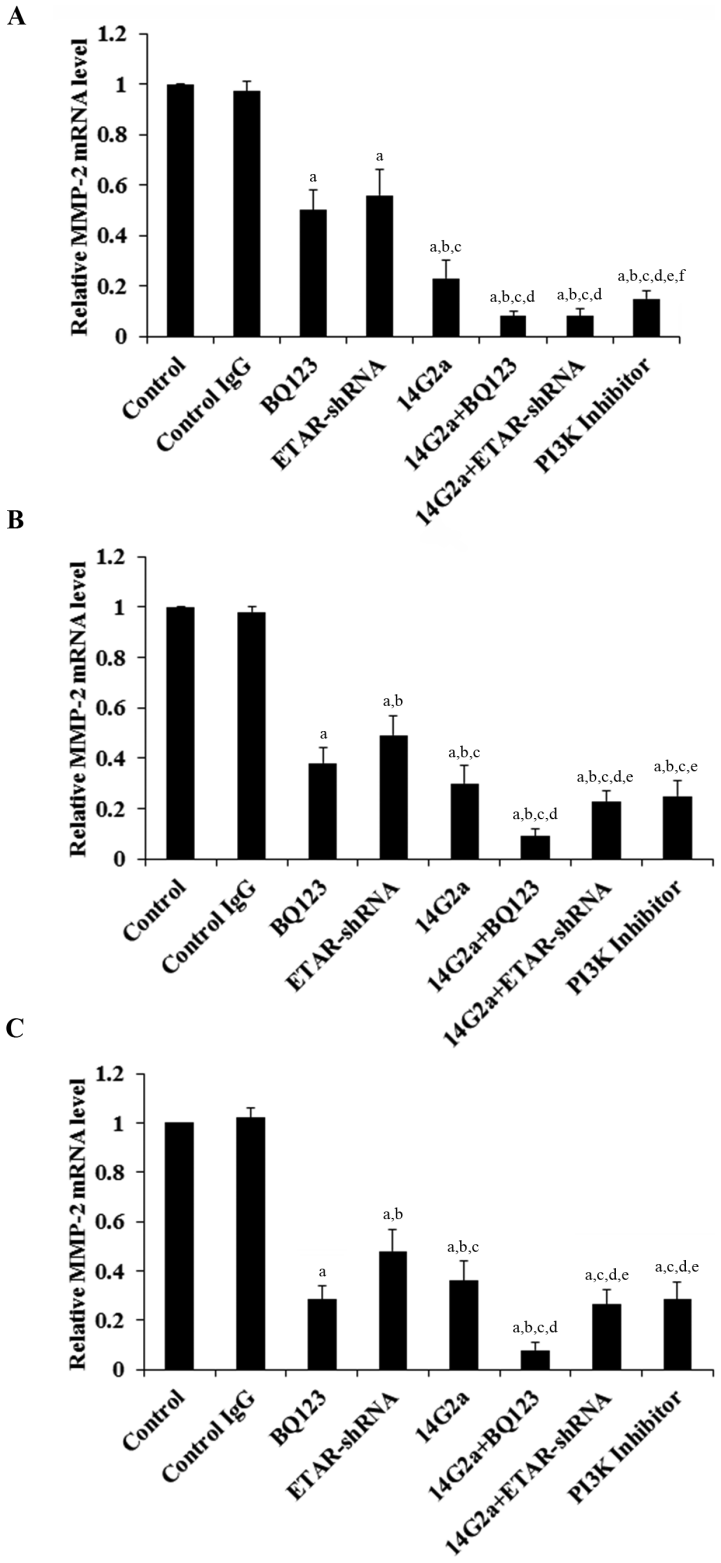
Effects of anti-ganglioside GD2 14G2a monoclonal antibody (mAb) alone or in combination with ET A receptor (ETAR) antagonist on matrix metalloproteinase-2 (MMP-2) mRNA levels in osteosarcoma (OS) cells. MMP-2 mRNA levels were determined by real-time RT-PCR in Saos-2 (*A*), MG-63 (*B*) and SJSA-1 (*C*) OS cells treated with control IgG (PK136 mAb, 50 µg/mL), 14G2a mAb (50 µg/mL), selective ETAR antagonist BQ123 (5 µM), and 14G2a (50 µg/mL)+BQ123 (5 µM) for 48 hours. Cells with knockdown of ETAR (ETAR-shRNA) with or without 14G2a mAb treatment were also tested. Cells treated with selective phosphatidylinositide 3-kinase (PI3K) inhibitor BKM120 (50 µM) was used as a positive control. The MMP-2 mRNA level was shown as fold changes to that of the untreated control cells (designated as 1). Each experiment was repeated for three times in duplicates. Data values were expressed as Mean+SD. ^a^ *p*<0.05 vs. control or control IgG; ^b^ *p*<0.05 vs. BQ123; ^c^ *p*<0.05 vs. ETAR-shRNA; ^d^ *p*<0.05 vs. 14G2a; ^e^ *p*<0.05 vs. 14G2a+BQ123; ^f^ *p*<0.05 vs. 14G2a+ETAR-shRNA.

**Figure 4:**
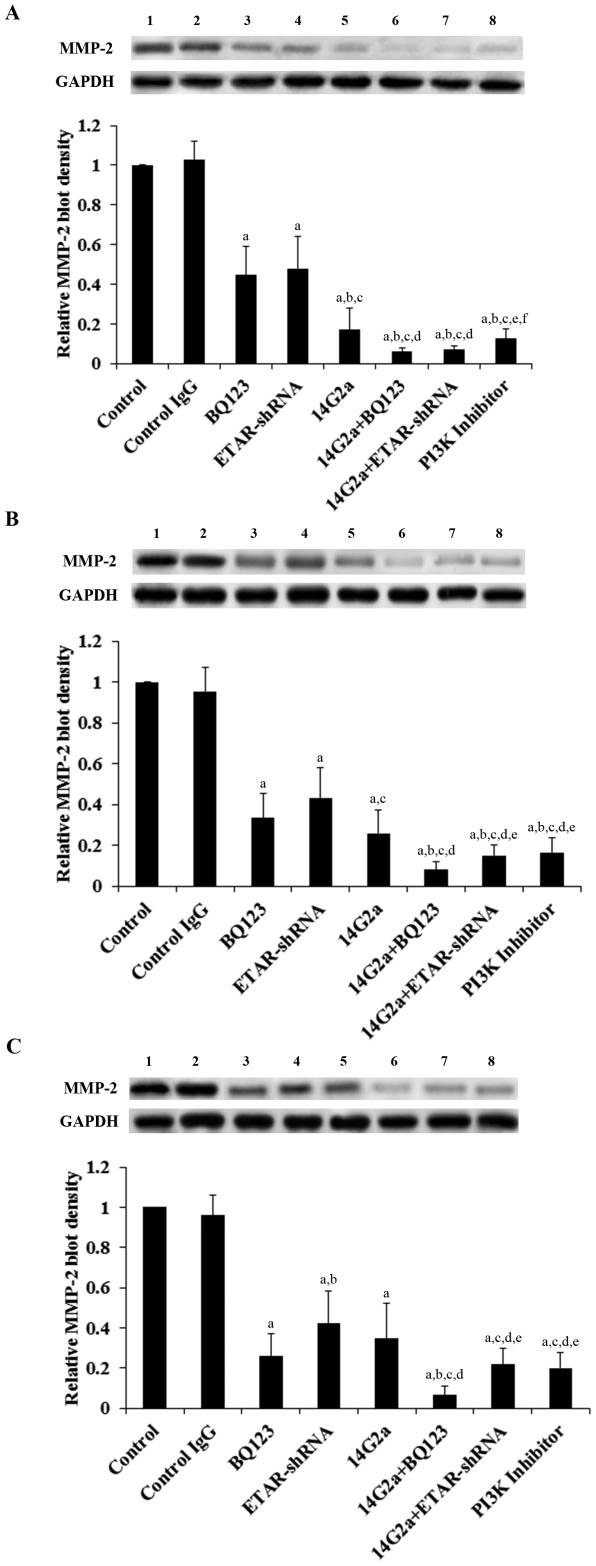
Effects of anti-ganglioside GD2 14G2a monoclonal antibody (mAb) alone or in combination with ET A receptor (ETAR) antagonist on matrix metalloproteinase-2 (MMP-2) protein levels in osteosarcoma (OS) cells. MMP-2 protein levels were determined by Western blot analyses in in Saos-2 (*A*), MG-63 (*B*) and SJSA-1 (*C*) cells treated with control IgG (50 µg/mL, lane 2), selective ETAR antagonist BQ123 (5 µM, lane 3), stably-transduced ETAR-shRNA (lane 4), 14G2a mAb (50 µg/mL, lane 5), 14G2a+BQ123 (lane 6), 14G2a+ETAR-shRNA (lane 7), and selective phosphatidylinositide 3-kinase (PI3K) inhibitor BKM120 (50 µM, lane 8). The untreated control was in *lane 1*. Glyceraldehyde-3-phosphate dehydrogenase (GAPDH) blotting was used as a loading control. Density of the MMP-2 blot was normalized against that of GAPDH to obtain a relative blot density, which was expressed as fold changes to the relative MMP-2 blot density of the untreated control cells (designated as 1). Three independent experiments were performed for each Western blot analysis. Data values were expressed as Mean+SD. ^a^ *p*<0.05 vs. control or control IgG; ^b^ *p*<0.05 vs. BQ123; ^c^ *p*<0.05 vs. ETAR-shRNA; ^d^ *p*<0.05 vs. 14G2a; ^e^ *p*<0.05 vs. 14G2a+BQ123; ^f^ *p*<0.05 vs. 14G2a+ETAR-shRNA.

**Figure 5:**
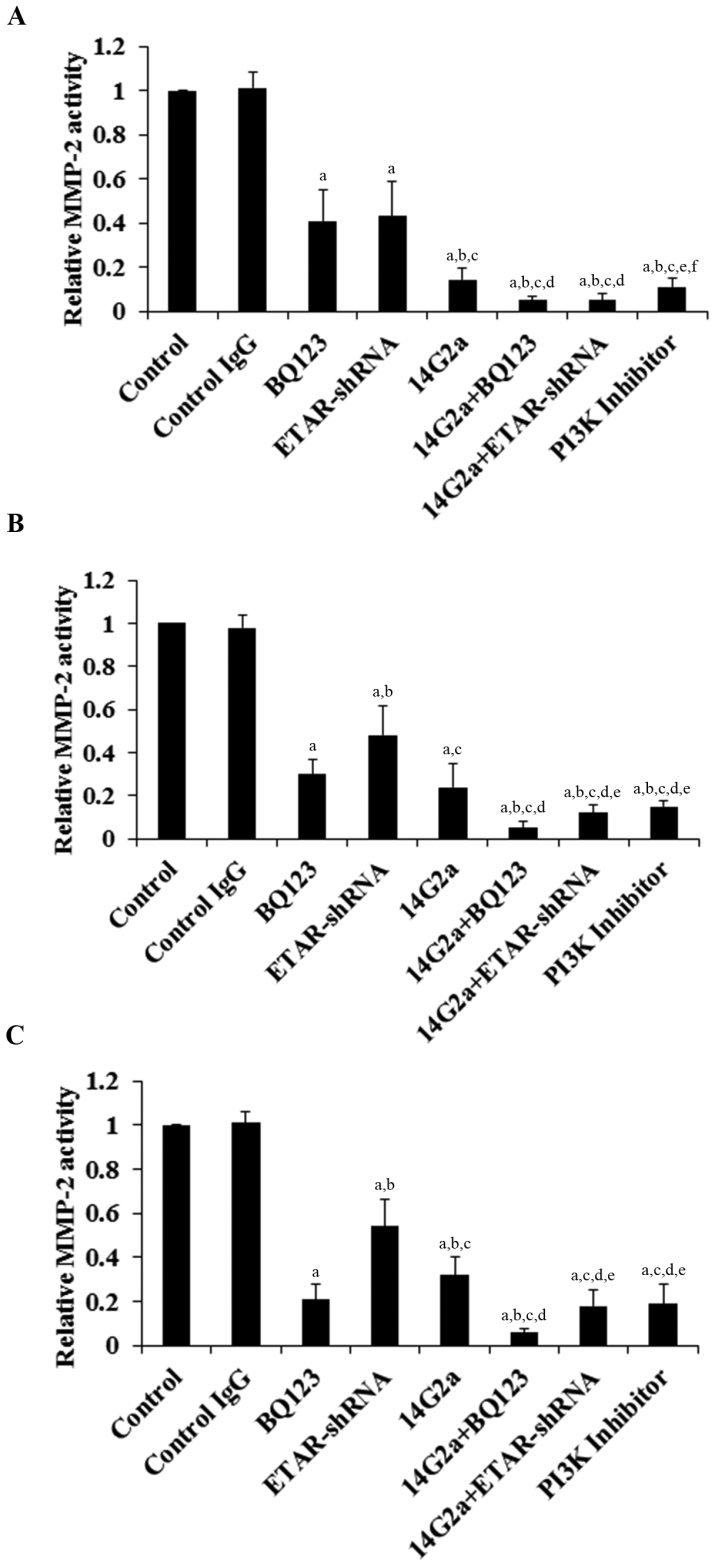
Effects of anti-ganglioside GD2 14G2a monoclonal antibody (mAb) alone or in combination with ET A receptor (ETAR) antagonist on matrix metalloproteinase-2 (MMP-2) activity in osteosarcoma (OS) cells. MMP-2 activities were determined with a SensoLyte 520 MMP-2 Assay Kit (AnaSpec) in Saos-2 (*A*), MG-63 (*B*) and SJSA-1 (*C*) OS cells treated with control IgG (PK136 mAb, 50 µg/mL), 14G2a mAb (50 µg/mL), selective ETAR antagonist BQ123 (5 µM), and 14G2a (50 µg/mL)+BQ123 (5 µM) for 48 hours. Cells with knockdown of ETAR (ETAR-shRNA) with or without 14G2a mAb treatment were also tested. Cells treated with selective phosphatidylinositide 3-kinase (PI3K) inhibitor BKM120 (50 µM) was used as a positive control. The MMP-2 activity was shown as fold changes to that of the untreated control cells (designated as 1). Each experiment was repeated for three times in duplicates. Data values were expressed as Mean+SD. ^a^ *p*<0.05 vs. control or control IgG; ^b^ *p*<0.05 vs. BQ123; ^c^ *p*<0.05 vs. ETAR-shRNA; ^d^ *p*<0.05 vs. 14G2a; ^e^ *p*<0.05 vs. 14G2a+BQ123; ^f^ *p*<0.05 vs. 14G2a+ETAR-shRNA.

MMPs play a critical role in cancer cell invasion [12]. Among different MMPs tested, we found that the MMP-2 expression at both the mRNA (Fig. 3) and the protein levels (Fig. 4) was significantly altered by ETAR antagonism and 14G2a mAb alone or in combination. BQ123, ETAR-shRNA and 14G2a mAb individually decreased the MMP-2 mRNA (Fig. 3) and protein expression (Fig. 4). 14G2a mAb in combination with BQ123 or ETAR-shRNA showed significantly stronger inhibitory effects compared with each individual treatment. In all three cell lines tested, 14G2a mAb in combination with BQ123 showed the strongest inhibitory effect on MMP-2 expression at both the mRNA and the protein levels in OS cells. Similar data trend was observed with the MMP-2 activity (Fig. 5).

### Effects of anti-ganglioside GD2 14G2a mAb alone or in combination with ETAR antagonist on OS cell viability

**Figure 6:**
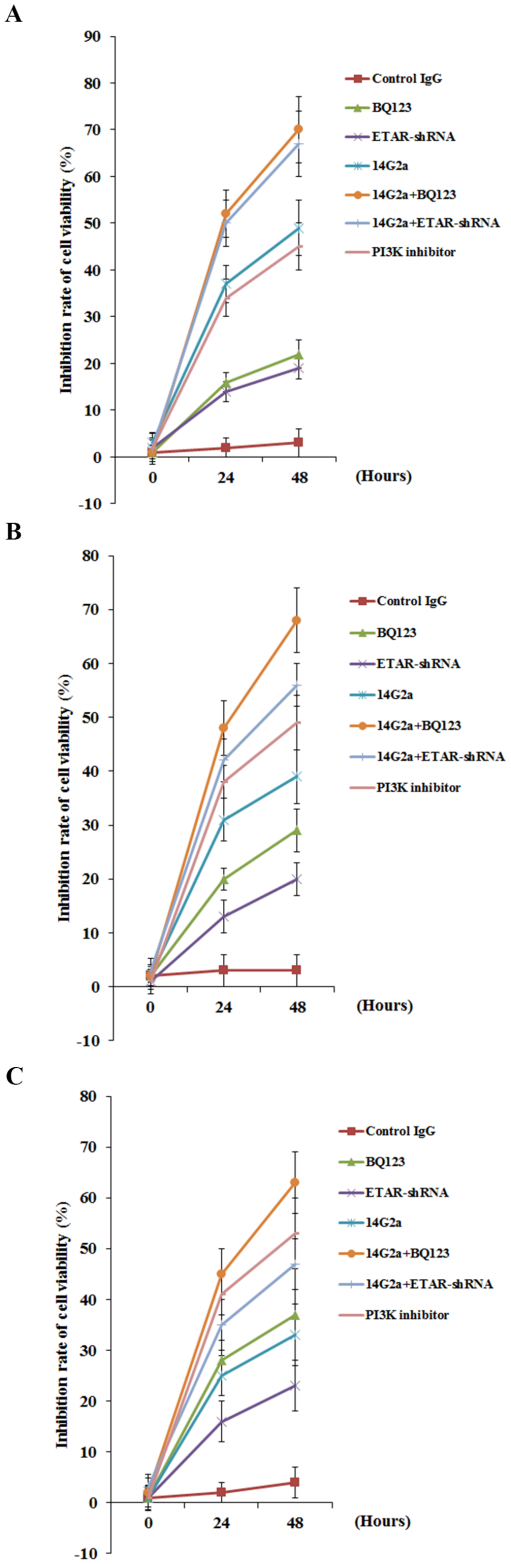
Effects of anti-ganglioside GD2 14G2a monoclonal antibody (mAb) alone or in combination with ET A receptor (ETAR) antagonist on osteosarcoma (OS) cell viability. Methlythiazoletetrazolium (MTT) cell viability assays were performed in Saos-2 (*A*), MG-63 (*B*) and SJSA-1 (*C*) OS cells treated with control IgG (PK136 mAb, 50 µg/mL), 14G2a mAb (50 µg/mL), selective ETAR antagonist BQ123 (5 µM), and 14G2a (50 µg/mL)+BQ123 (5 µM) for 24 or 48 hours. Cells with knockdown of ETAR (ETAR-shRNA) with or without 14G2a mAb treatment were also tested. Cells treated with selective phosphatidylinositide 3-kinase (PI3K) inhibitor BKM120 (50 µM) was used as a positive control. Viability of the control cells was designated as 100%. The inhibition rate of cell viability was calculated and shown as a percentage of the control cell viability. Each experiment was repeated for three times in triplicates. Data values were expressed as Mean+SD.

14G2a mAb reportedly decreases neuroblastoma cell viability [8], and ET-1/ETAR signaling has been shown to increase cancer cell proliferation and survival [26]. Thus, we next explored the effects of 14G2a mAb alone or in combination with BQ123 on OS cell viability. As shown in Fig. 6, inhibition of cell viability in Saos-2 (*A*), MG-63 (*B*) and SJSA-1 (*C*) cells treated with control IgG (PK136 mAb, 50 µg/mL), 14G2a mAb (50 µg/mL), selective ETAR antagonist BQ123 (5 µM), and 14G2a (50 µg/mL)+BQ123 (5 µM) were measured at 24 and 48 hours after treatment, respectively. Cells with knockdown of ETAR (ETAR-shRNA) with or without 14G2a mAb treatment were also tested. Cells treated with selective PI3K inhibitor BKM120 (50 µM) was used as a positive control. Fig. 6 shows that BQ123, ETAR-shRNA and 14G2a mAb individually decreased OS cell viability. 14G2a mAb in combination with BQ123 or ETAR-shRNA showed significantly stronger inhibitory effects compared with each individual treatment. In all three cell lines tested, 14G2a mAb in combination with BQ123 showed the strongest inhibitory effect on OS cell viability.

### Effects of anti-ganglioside GD2 14G2a mAb alone or in combination with ETAR antagonist on PI3K activity and phosphorylation of Akt in OS cells

**Figure 7:**
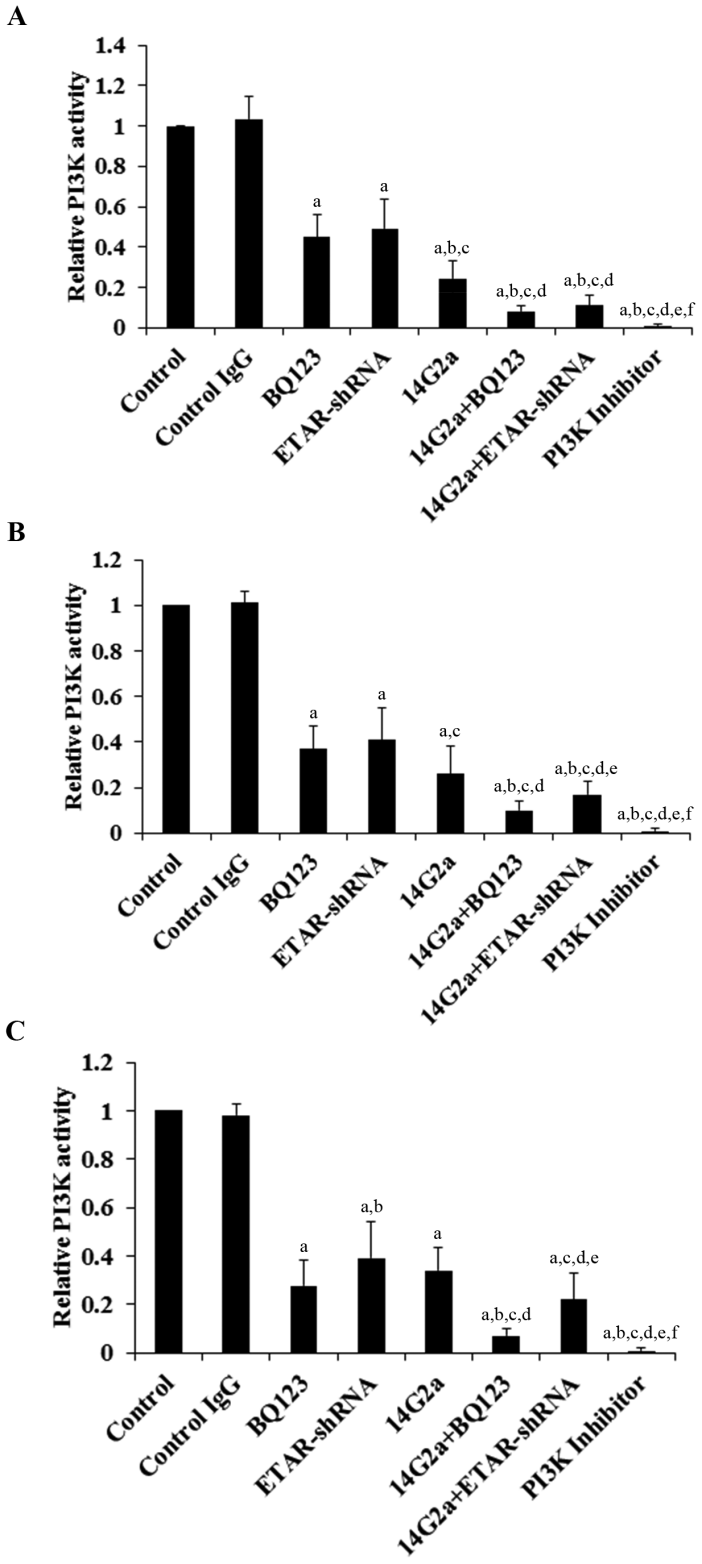
Effects of anti-ganglioside GD2 14G2a monoclonal antibody (mAb) alone or in combination with ET A receptor (ETAR) antagonist on phosphatidylinositide 3-kinase (PI3K) activity in osteosarcoma (OS) cells. PI3K activities were determined with a PI3K Activity ELISA kit (Echelon Biosciences) in Saos-2 (*A*), MG-63 (*B*) and SJSA-1 (*C*) OS cells treated with control IgG (PK136 mAb, 50 µg/mL), 14G2a mAb (50 µg/mL), selective ETAR antagonist BQ123 (5 µM), and 14G2a (50 µg/mL)+BQ123 (5 µM) for 48 hours. Cells with knockdown of ETAR (ETAR-shRNA) with or without 14G2a mAb treatment were also tested. Cells treated with selective phosphatidylinositide 3-kinase (PI3K) inhibitor BKM120 (50 µM) was used as a positive control. The PI3K activity was shown as fold changes to that of the untreated control cells (designated as 1). Each experiment was repeated for three times in duplicates. Data values were expressed as Mean+SD. ^a^ *p*<0.05 vs. control or control IgG; ^b^ *p*<0.05 vs. BQ123; ^c^ *p*<0.05 vs. ETAR-shRNA; ^d^ *p*<0.05 vs. 14G2a; ^e^ *p*<0.05 vs. 14G2a+BQ123; ^f^ *p*<0.05 vs. 14G2a+ETAR-shRNA.

**Figure 8:**
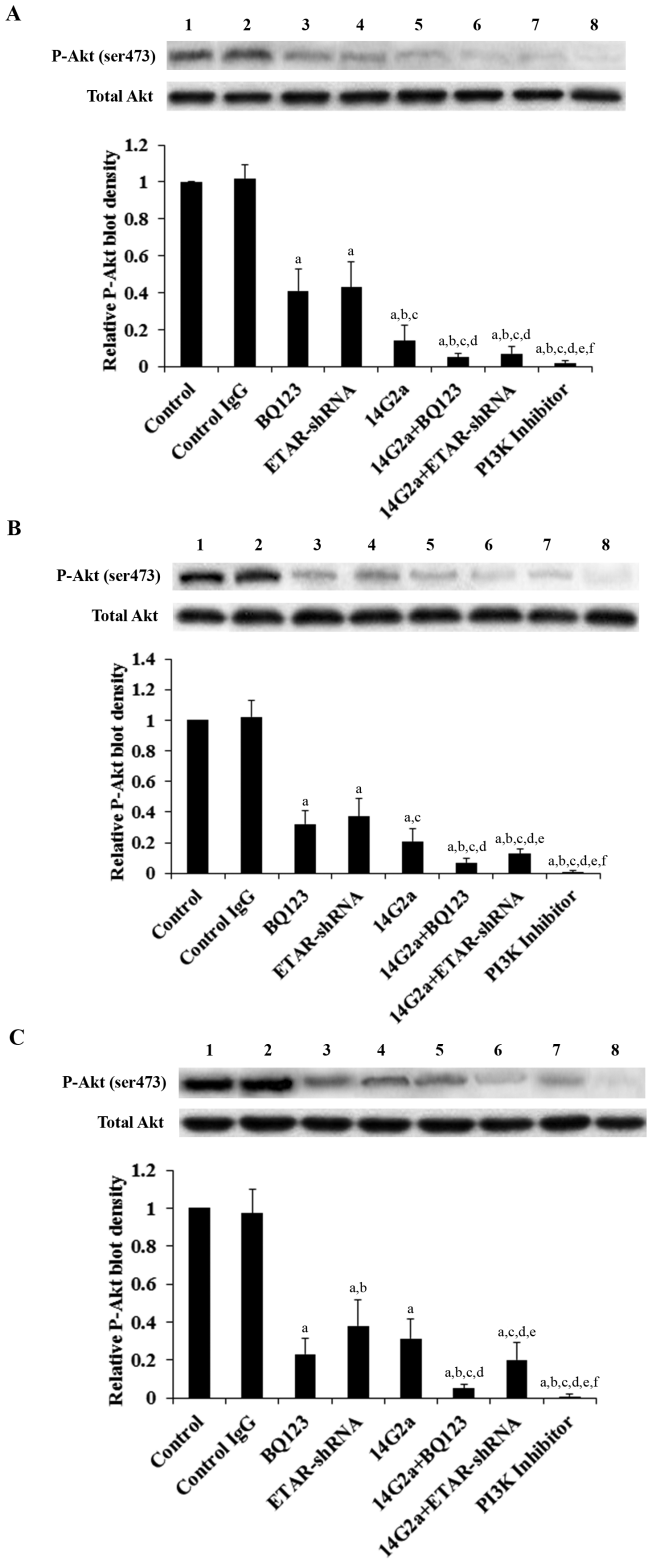
Effects of anti-ganglioside GD2 14G2a monoclonal antibody (mAb) alone or in combination with ET A receptor (ETAR) antagonist on phosphorylated Akt (P-Akt) level in osteosarcoma (OS) cells. Levels of total Akt and P-Akt at serine 473 (ser473) were determined by Western blot analyses in Saos-2 (*A*), MG-63 (*B*) and SJSA-1 (*C*) cells treated with control IgG (50 µg/mL, lane 2), selective ETAR antagonist BQ123 (5 µM, lane 3), stably-transduced ETAR-shRNA (lane 4), 14G2a mAb (50 µg/mL, lane 5), 14G2a+BQ123 (lane 6), 14G2a+ETAR-shRNA (lane 7), and selective phosphatidylinositide 3-kinase (PI3K) inhibitor BKM120 (50 µM, lane 8). The untreated control was in *lane 1*. Density of the P-Akt (ser473) blot was normalized against that of total Akt to obtain a relative blot density, which was expressed as fold changes to the relative P-Akt (ser473) blot density of the untreated control cells (designated as 1). Three independent experiments were performed for each Western blot analysis. Data values were expressed as Mean+SD. ^a^ *p*<0.05 vs. control or control IgG; ^b^ *p*<0.05 vs. BQ123; ^c^ *p*<0.05 vs. ETAR-shRNA; ^d^ *p*<0.05 vs. 14G2a; ^e^ *p*<0.05 vs. 14G2a+BQ123; ^f^ *p*<0.05 vs. 14G2a+ETAR-shRNA.

Both ET-1/ETAR signaling and ganglioside GD2 reportedly can activate the PI3K/Akt pathway in cancer cells [17], [18]. We next examined the effects of 14G2a mAb alone or in combination with BQ123 on the PI3K activity and phosphorylation of Akt in OS cells. In OS cells treated for 48 hours, BQ123, ETAR-shRNA and 14G2a mAb individually decreased the PI3k activity (Fig. 7) and phosphorylation at serine 473 (ser473) of Akt (Fig. 8), which is required for full activation of Akt. 14G2a mAb in combination with BQ123 or ETAR-shRNA showed significantly stronger inhibitory effects compared with each individual treatment. In all three cell lines tested, 14G2a mAb in combination with BQ123 showed the strongest inhibitory effect on the PI3K activity and ser473 phosphorylation of Akt in OS cells.

We noted a consistent data pattern in all the above experiments: The inhibitory effects of BQ123 on cell invasiveness (Fig. 2), MMP-2 expression (Fig. 3 and Fig. 4) and activity (Fig. 5), cell viability (Fig. 6), PI3k activity (Fig. 7), and phosphorylation/activation of Akt (Fig. 8) in OS cells was increasing with the cellular ETAR level (Saos-2<MG-63<SJSA-1), while the inhibitory effect of 14G2a mAb was decreasing with the cellular ETAR level (Saos-2>MG-63>SJSA-1). 14G2a in combination with BQ123 showed the strongest inhibitory effects, which were comparable among the cell lines.

## Discussion

Ganglioside GD2 has been associated with enhanced cancer cell viability and invasive activity [5]. There has been a growing number of evidence that GD2-specific antibodies may exhibit anti-viability effects independent of the immune system [7]. A recent study indicates that ganglioside GD2 is highly expressed on OS, and suggests that clinical trials are needed to assess the efficacy of targeting GD2 in patients with OS [9]. On the other hand, the role of ET-1/ETAR signaling in OS progression has been well established [10]–[12]. In the present study, we for the first time demonstrate the inhibitory effects of a GD2-specific mAb alone or in combination with ETAR antagonist on OS cell invasiveness and viability.

Among different anti-GD2 mAbs that have been reported to show antitumor effects [7], [8], [27], only 14G2a showed significant effects on OS cell invasion and viability in our pilot studies and thus was employed in this study. Human OS cell lines Saos-2, MG-63 and SJSA-1 were used in this study, because they reportedly express ET-1 and ETAR [11]. In agreement with a previous study [11], while the ET-1 levels in the three cell lines were comparable, there were significant differences in the ETAR expression level, in the order of Saos-2<MG-63<SJSA-1. In addition to blocking ET-1/ETAR signaling with BQ123, we also knocked down ETAR in the cell lines to verify the role of ETAR in OS invasiveness and viability. As the knockdown rate of endogenous ETAR was about 75% and the cell lines had significant differences in the expression level of ETAR, the ETAR amount remaining in the cells after ETAR-shRNA transduction was in the order of Saos-2<MG-63<SJSA-1, which may explain for the decreasing inhibitory effects of ETAR-shRNA or 14G2a+ETAR-shRNA on OS cell invasiveness and viability in the order of Saos-2>MG-63>SJSA-1. In contrast, BQ123 in a saturation concentration (5 µM) for all three cell lines showed increasing inhibitory effects on OS cell invasiveness and viability in the order of Saos-2<MG-63<SJSA-1, likely due to its inhibition of the increasing constitutive ET-1/ETAR signaling in the cells.

While the inhibitory effect of BQ123 on OS cell invasiveness, MMP-2 expression and activity, cell viability, PI3k activity, and phosphorylation/activation of Akt in OS cells was increasing with the cellular ETAR level (Saos-2<MG-63<SJSA-1), the inhibitory effect of 14G2a mAb was decreasing with the cellular ETAR level (Saos-2>MG-63>SJSA-1). Combined treatment with 14G2a and BQ123 showed the strongest inhibitory effects, which were comparable among the cell lines. The findings suggest that constitutive ET-1/ETAR signaling may antagonize the effect of 14G2a mAb on OS cell invasiveness and viability, which explains why blocking ET-1/ETAR signaling with BQ123 markedly enhanced the inhibitory effects of 14G2a mAb.

MMPs play a critical role in cancer cell invasion [12]. Among different MMPs tested, the MMP-2 expression was significantly altered by BQ123, ETAR-knockdown and 14G2a mAb alone or in combination, which displayed similar data trend as that in the MMP-2 activity and OS cell invasiveness. The PI3K/Akt pathway reportedly can stimulate MMP-2 expression [28], [29]. As we found that ETAR antagonism and anti-GD2 mAb markedly inhibited the PI3K/Akt pathway alone or in combination in OS cells, it is likely that ETAR antagonism and anti-GD2 mAb alone or in combination inhibit OS cell invasiveness by down-regulating the MMP-2 expression/activity mainly through inhibiting the PI3K/Akt pathway.

The PI3K/Akt pathway reportedly plays an important role in OS cell invasiveness and viability [13]–[16]. ET-1 has been reported to activate the PI3K/Akt pathway via the ETAR [17]. The PI3K/Akt pathway is also involved in ganglioside GD2-induced tumorigenicity and aggressiveness of breast cancer cells [18]. Thus, we included cells treated with selective PI3K inhibitor BKM120 (50 µM) as a positive control in OS cell invasiveness and viability experiments [25]. In this study, BKM120 significantly inhibited OS cell invasiveness and viability, in agreement with previous studies [13]–[16]. 14G2a mAb, BQ123, or ETAR-shRNA individually decreased the PI3K activity in OS cells, which was reflected in ser473 phosphorylation of Akt. 14G2a mAb in combination with BQ123 or ETAR-shRNA showed significantly stronger inhibitory effects on the PI3K activity and ser473 phosphorylation of Akt compared with each individual treatment. In view of the important role of the PI3K/Akt pathway in OS cell invasiveness and viability [13]–[16], our findings suggest that inhibiting PI3K/Akt is a common pathway for both ETAR antagonism and anti-GD2 mAb to inhibit OS cell invasiveness and viability. Nevertheless, as combined treatment with BQ123 and 14G2a showed stronger inhibitory effects than the PI3K inhibitor, other pathways reportedly activated by ET-1-/ETAR signaling and anti-GD2 mAb (such as mitogen-activated protein kinase and aurora A kinase pathways, respectively) may also be involved. Future studies are needed to elaborate this issue.

In conclusion, we provide the first in vitro evidence that anti-ganglioside GD2 14G2a mAb effectively inhibits cell invasiveness, MMP-2 expression and activity, and cell viability in human OS cells. ETAR antagonist BQ123 significantly enhances the inhibitory effects of 14G2a mAb, likely mainly through inhibiting the PI3K/Akt pathway. This study adds novel insights into OS treatment, which will serve as a solid basis for future in vivo studies on the effects of combined treatment of OS with anti-ganglioside GD2 mAbs and ETAR antagonists.
